# Effects of a biomechanical-based Tai Chi program on gait and posture in people with Parkinson’s disease: study protocol for a randomized controlled trial

**DOI:** 10.1186/s13063-023-07146-x

**Published:** 2023-06-30

**Authors:** Nok-Yeung Law, Jing Xian Li, Qingguang Zhu, Julie Nantel

**Affiliations:** 1 Schools of Human Kinetics, Faculty of Health Sciences, University of Ottawa, Ottawa, Canada; 2 Research Institute of Tuina, Shanghai University of Traditional Chinese Medicine, Shanghai, China

**Keywords:** Parkinson’s disease, Gait, Postural stability, Falls, Tai Chi

## Abstract

**Background:**

Parkinson’s disease (PD) is associated with changes in gait and posture, which increases the rate of falls and injuries in this population. Tai Chi (TC) training enhances the movement capacity of patients with PD. However, the understanding of the effect of TC training on gait and postural stability in PD is lacking. This study aims to examine the effect of biomechanical-based TC training on dynamic postural stability and its relationship with walking performance.

**Methods/design:**

A single-blind, randomized control trial of 40 individuals with early-stage PD was conducted (Hoehn and Yahr stages 1 to 3). Patients with PD will be randomly assigned to either the TC or control group. The TC group will participate in a biomechanical-based TC training program that is formed based on the movement analysis of TC and will be practiced thrice a week for 12 weeks. The control group will be required to engage in at least 60 min of regular physical activity (PA) on their own for three times per week for 12 weeks. The primary and secondary outcomes will be assessed at baseline and at 6 and 12 weeks after commencing the study protocol. The primary outcome measures will include dynamic postural stability indicated by the center of mass and center of pressure separation distance and clearance distance of the heel and toe measured during fixed-obstacle crossing. The secondary measures are gait speed, cadence, step length during level surface walking (simple task), and fixed-obstacle crossing (challenging task). The Unified Parkinson’s Disease Rating Scale, single leg-stance test with eyes open and closed, and three cognitive scores (Stroop Test, Trail Making Test Part B, and the Wisconsin Card Sorting Test) were also employed.

**Discussion:**

This protocol could lead to the development of a biomechanics TC training program for the improvement of gait and postural stability among individuals with PD. The program could enhance the understanding of the effect of TC training on gait and postural stability and could help improve or preserve the postural stability, self-confidence, and active participation in social activities of the participants, thus enhancing their overall quality of life.

**Trial registration:**

ClinicalTrials.gov NCT04644367. Registered on 25 November 2020.

**Supplementary Information:**

The online version contains supplementary material available at 10.1186/s13063-023-07146-x.

## Administrative information

Note: the numbers in curly brackets in this protocol refer to SPIRIT checklist item numbers. The order of the items has been modified to group similar items (see http://www.equator-network.org/reporting-guidelines/spirit-2013-statement-defining-standard-protocol-items-for-clinical-trials/).

**Table:** 

Title {1}	Effects of a biomechanical-based Tai Chi program on gait and posture in people with Parkinson’s disease: study protocol for a randomized controlled trial
Trial registration {2a and 2b}.	NCT04644367, ClinicalTrials.gov
Protocol version {3}	2020–2-19. Version 1.
Funding {4}	This study was not supported by any funding.
Author details {5a}	Nok-Yeung Law^1*^, Jing Xian Li^1*^, Qingguang Zhu^2*^, Julie Nantel^1^ ^*^, Correspondence author ^1^, Schools of Human Kinetics, Faculty of Health Sciences, University of Ottawa, Canada ^2^, Research Institute of Tuina, Shanghai University of Traditional Chinese Medicine, Shanghai, China
Name and contact information for the trial sponsor {5b}	This trial was supported by the University of Ottawa.
Role of sponsor {5c}	The sponsors did not play a role in the design of the study and collection, analysis, and interpretation of data and in writing the manuscript.

## Introduction

### Background and rationale {6a}

Parkinson’s disease (PD) is a growing concern in Canada, where approximately 100,000 people are living with PD; this number is expected to rise to 163,700 by 2031 [1]. The direct and indirect socioeconomic costs in Canada associated with this neurological condition are estimated to be more than $1.214B [2]. Postural instability and gait difficulties are common problems reported among people with PD [3, 4]. People with PD have difficulties with daily tasks such as walking caused by gait-related changes in step width and step length that can lead to reduced walking speeds. This “cautious gait” approach is similar to older adults who are at a high risk of falls [5, 6]. Falls and the loss of the ability to live independently are among the key concerns of people with PD. A 1-year follow-up study among 109 subjects with idiopathic PD diagnosed according to the brain bank criteria reported a fall occurrence of 68.3% [7].

Exercise intervention programs are being used in conjunction with drug therapy (such as levodopa, carbidopa) as standard of care to manage the symptoms related to PD [8, 9]. Tai Chi (TC) is a body-mind exercise that has been recommended by *Parkinson’s Canada* as one of the few exercise programs specific for those with PD [10]. TC training is suggested to help improve the awareness of whole-body movement and joint proprioception and increase muscle strength and endurance of the lower limb, thus strengthening the neuromuscular reaction of muscles in the lower extremities [11, 12]. For people with PD, TC training would help in challenging postural stability, particularly in the mediolateral (M-L) direction that is closely related to fall prevention. It will also strengthen the muscles at the hip involved in postural control. Regular TC practice and training would lead to improvements in gait and postural stability by encouraging weight shifting, control displacement of the COM over the base of support, and comfort with stepping in different directions.

### Objectives {7}

Several TC studies for PD have been published [13, 14]. However, details about how TC programs were formulated or how TC forms were selected are unknown. This missing information may affect the training effect because TC has many forms and has different styles. Moreover, the effect of TC training on dynamic postural stability and how it relates to walking performance and challenging walking, such as obstacle crossing, is unclear. This information gap can be difficult for people with PD, because dynamic postural stability is challenged. This study aims to examine the impact of a biomechanics-based TC program training on gait and postural stability, particularly on dynamic postural stability and how it relates to walking performance and obstacle crossing. Based on our previous studies [11, 15, 16], it is hypothesized that a biomechanics-based TC program training will help in improving gait and dynamic postural stability by increasing walking speed, stability in mediolateral (M-L) direction, and toe and heel clearance distance during obstacle crossing in the individuals with PD. This protocol could lead to the development of an innovative balance and gait training program tailored to individuals with early-stage PD.

### Trial design {8}

**Fig. 1:**
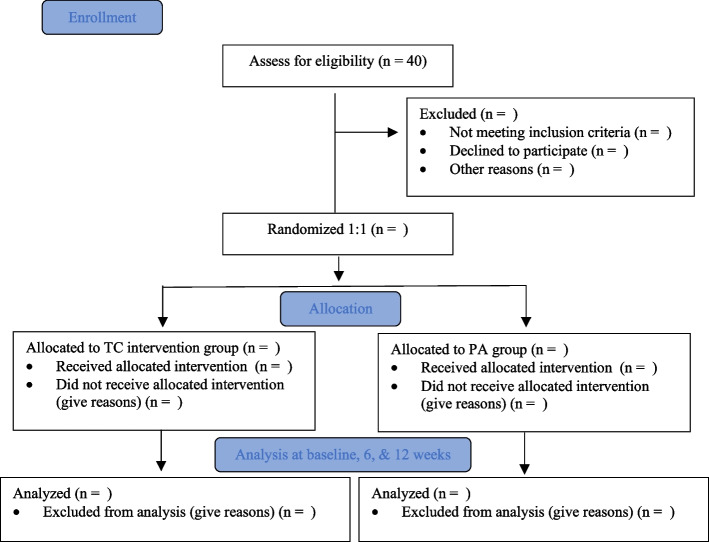
Flow diagram of TC study with design and illustration of experimental and control groups’ allocation

A single-blind randomized control trial will be conducted to compare the effects of a TC program with a physical activity (PA) routine (Fig. 1). The study will be conducted for 12 weeks (3 months), and this period will include a 12-week intervention period with primary and secondary outcomes measured at baseline and at 6 and 12 weeks.

## Methods: participants, interventions, and outcomes

### Study setting {9}

University of Ottawa’s Human Movement Biomechanics Laboratory will serve as the primary site for the study. TC interventions will be offered at community centers in Ottawa, Canada. The PA routine can be performed inside or outside the home environment.

### Eligibility criteria {10}

Participants will be included in the study if they meet the following criteria:

1. Have been diagnosed with PD and demonstrate a disease severity ranging from 1 to 3 on the Hoehn and Yahr (H&Y) scale
2. Have no fluctuations in motor symptoms as reported by the motor section of the Unified Parkinson’s Disease Rating Scale (UPDRS-III)
3. Have stable PD (such as levodopa or carbidopa) and doctor-prescribed medication use, that is, fluctuation did not occur at the time of medication intake
4. Can stand and walk independently
5. Available thrice a week over a period of 12 weeks
6. Be able to engage in PA for at least 60 min on their own, thrice per week, without prior experience practicing TC regularly

Participants will be excluded from the study if any of the following conditions are reported:

1. Enrolled into any other behavioral or pharmacological studies
2. Have a Montreal Cognitive Assessment (MoCA) score of < 26/27 (that indicates some mild cognitive impairment (MCI)) [17]
3. Have had a serious medical condition or uncontrolled hypertension (equal or greater than a systolic 180 or diastolic 110 reading)
4. Have any debilitating conditions that could prevent them from performing any PA for a minimum of 60 min
5. Have practiced or have experience of practicing TC in an organized class or program within the last 5 years at the start of participation in the study

### Who will take informed consent? {26a}

The primary investigator will obtain informed consent from the potential trial participants.

### Additional consent provisions for collection and use of participant data and biological specimens {26b}

No secondary participant information or data will be collected, nor any biological specimens will be collected.

### Interventions

#### Explanation for the choice of comparators {6b}

The two comparators are the TC intervention and PA group (control group). The two comparators were chosen to understand the effect of 12-week biomechanical-based TC training on the gait and posture of people with PD compared with those who will maintain their regular PA. An active PA (control) group was chosen for avoiding an unfair comparison to participants who are sedentary or who are inactive.

#### Intervention description {11a}

## TC program development

**Fig. 2:**
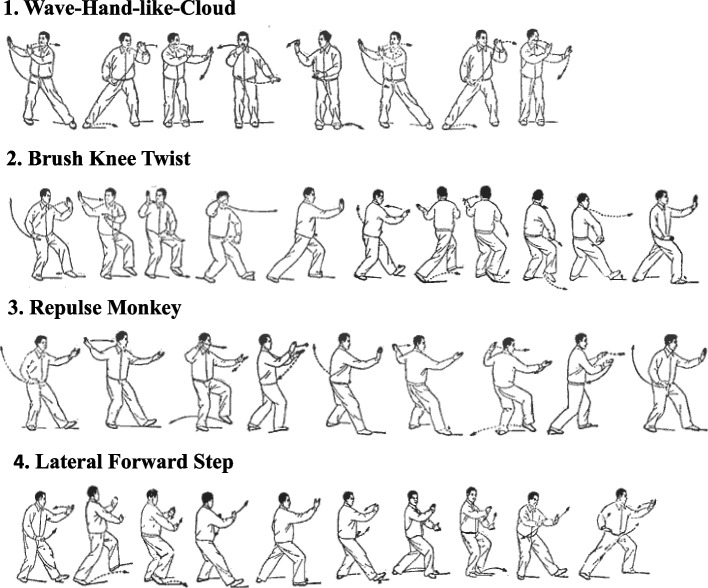
Illustration of four dynamic forms from the proposed 7-form TC program

The biomechanical-based TC training program has been developed based on research with an experienced TC master, our previous work, and by biomechanical analysis of TC movement. Seven TC movements were chosen based on the following considerations:

1. Easy to learn.
2. Feasible to practice.
3. Can be practiced either individually or in groups.
4. Space requirement is minimal (e.g., practicing inside the home, living room, or yard).
5. The seven selected TC movements and the three static and four dynamic forms are listed in Table 1. A static TC movement was defined as the movement in which the center of mass (COM) was maintained within the limits of the body’s base of support (BOS), whereas a dynamic TC movement was defined as the movement, in which the body’s COM was outside the limits of the body’s BOS. Figure 2 illustrates three dynamic TC movements from the seven TC movements. These TC movements are typical TC movements and are present in all classic TC styles, such as Wu, Yang, and Chen styles [18]. The biomechanical analysis of the seven selected TC movements in terms of their kinematics, kinetics, and muscle activities demonstrates their unique features compared with walking. These movements require increased foot clearance, step length, and movement in the backward, sideway, 45° angle, and forward directions [15, 19]. Regular performance of these movements would result in special training to postural control, particularly in the adductor and tensor fasciae latae muscles that are responsible for maintaining M-L postural stability and assisting in preventing falls [15, 16]. TC training can help in challenging M-L stability through shifting of the body’s COM, using the full range of motion of joints, and increasing step width [15, 16]. Electromyography activity while performing the seven TC forms has revealed a higher activation of the hip adductor muscles compared with walking, suggesting that good muscle training prevents lateral falls [18].

**Table 1:** 7-form TC program for people with PD

TC form	Characteristics
Form 1: Starting form	Standing (arms): arms from down to up
Form 2: Hero lift the sky	Standing (arms): push up to down
Form 3: Standing, push hand back	Standing (arms): neutral to back
Form 4: Wave-hand-like-cloud	Sideway stepping: left and right
Form 5: Brush knee twist step	Forward diagonal stepping: left and right
Form 6: Repulse monkey	Backward diagonal stepping: left and right
Form 7: Lateral forward step	Lateral diagonal stepping: left and right

## TC training group

For the TC group, the first 2 weeks of the program will require the participants to work on some light breathing and simple upper-limb forms. The basic TC forms will be introduced with a focus on becoming comfortable with the flow and rhythm of the movements. Each session will run for 60 min and will be supervised by a TC instructor in a community center. The training will be gradual and range from low to moderate intensity. Participants will warm up for 5–10 min, perform core activities for 40 min, and cool down for 5 min. Three sessions will be offered weekly for 12 weeks; the participants will be asked to attend at least two sessions per week. The third to sixth week of the program will focus on incorporating the upper and lower limb into the routine. These classes will focus on practicing the seven TC forms sequentially. The six remaining weeks of the program will focus on repetitions and performance mastery of the basic seven-form routine. The participants will be allowed to practice TC at home. Any TC that the participant performs in their home or on their own time will be recorded by themselves by using a monitoring form that will be distributed to them and collected weekly. The TC instructor will be present at each of the session to monitor the participants’ progress and to ensure that the movements are being performed correctly.

## Physical activity group

The PA group will track their level of PA over 12 weeks. During this period, the participants will be required to engage in at least 60 min of regular PA on their own for three times per week. Regular PA may include walking, cleaning, performing chores inside the home, and/or climbing the stairs. No restriction will be made to limit other forms of PA. Individuals in the PA group will be permitted to engage in organized sports and instructor-led class such as boxing and dance to ensure that the participant recruitment process is feasible. An active control group was used for comparisons between the groups who will or will not receive formal TC training and to reflect the daily lifestyle and activities of a typical individual with PD from the community. Similar to the TC group, the participants in this group will be asked to complete an activity log form for monitoring, and this form will be collected weekly to monitor their regular PA levels.

### Criteria for discontinuing or modifying allocated interventions {11b}

At any point during the study, either during the intervention period or laboratory testing sessions, the participants will be free to refuse to participate. If he or she chooses to participate, the participant may withdraw from the study at any time for any reason.

### Strategies for improved adherence to interventions {11c}

For the group receiving the TC intervention, three class sessions will be offered weekly for 12 weeks. The participants will be asked to attend three sessions per week. These classes will be monitored by the PI and TC master. This set-up will ensure that the classes are offered at a flexible time for the participant that match their availability and schedules. The participants of the PA group will be asked to maintain regular PA levels and complete an activity log form for monitoring. This form will be collected weekly to monitor their regular PA levels to ensure that they will be motivated and consistent in their PA engagement and performance.

### Relevant concomitant care permitted or prohibited during the trial {11d}

No concomitant care and intervention requirements are either permitted or prohibited during the trial. The participants will be asked to maintain their medication dosage and any mandatory medical care or aid that they are receiving during the intervention period. To encourage engagement and enrollment into the study, we allowed the TC and PA groups to engage in forms of exercise and PA, and no restrictions will be made, because this set-up will not affect the outcome or results of the study.

### Provisions for post-trial care {30}

N/a.

#### Outcomes {12}

All outcome measures will be conducted by the researchers, blind to group allocation, after 1 week (baseline), 6 weeks (mid-intervention), and 12 weeks (post-intervention). The demographic information of the participants, including age, sex, and clinical information such as stage of PD and medication reported, will be collected from the participants.

### Primary outcome measures

## Dynamic postural stability

1. COM-COP separation distance in the anterior–posterior (A-P) and M-L directions. The separation distance of the body’s COM and COP will be determined through motion analysis of walking and obstacle crossing trials to reflect the body’s dynamic postural stability.
2. Clearance distance of the toe and heel. The clearance distance of the toe and heel will be measured from the position of the foot to the height of the obstacle during obstacle crossing. This parameter indicates the dynamic postural stability.

### Secondary outcome measures

## Gait performance

1. Gait speed. This is a measure of how quickly an individual can walk within a specified distance. Gait speed is a global measure of functional capacity, mobility, and movement performance.
2. Cadence. This measure is defined as the total number of full gait cycles that is completed over a period. In human movement analysis, this gait cycle is defined as the repeated instances of subsequent foot contacts of the same foot. Cadence can reflect the quality of the timing and frequency of the movement. A good stepping or walking cadence can reflect the individual’s movement capacity and their body’s ability to maintain rhythmicity during movement.
3. Step length. This measure is defined as the distance travelled by a person over one cycle from the initial heel strike to the next heel strike.

## Clinical and cognitive (Executive Function) assessment

1. Unified Parkinson’s Disease Rating Scale-motor examination (UPDRS-III). UPDRS-III is a standard clinical assessment tool for the evaluation of the severity and progression of the motor-related symptoms of PD. This scale has 14 items, each scored on a 5-point Likert scale ranging from 0 to 4, with 0 representing “no impairment” and 4 representing “marked impairment”.
2. Single-leg stance test with eyes open and closed. The participant will be asked to stand for as long as possible up to 60 s with either eyes open or closed. Trials will be terminated if the participant loses his or her balance, or if his or her feet move from the initial position. The sequence of the eyes open or closed trials will be randomized. Three trials will be performed with 3-min rest in between. This set-up was used, because this test can be used to reflect the muscle strength of the lower extremities and to test static postural stability [20].
3. Timed Up and Go Test (TUG). The individual will be instructed to stand up from a chair, walk 3 m as quick and safely as possible, cross a line marked on floor, turn around, walk back, and sit down. The individual will be permitted to use the chair’s arm or back support for assistance when standing up, if needed [21]. The time from the “go” signal until the completion will be recorded. This test aims to measure the functional mobility used in the clinical setting.
4. Trail Making Test Part B (TMT-B). The participants will be given a set of numbers and letters that they will be asked to connect with lines the numbers to letters in ascending order. The time consumed by the participant to complete this task will be recorded. TMT-B was used to measure processing speed and mental flexibility [22] and as a screening tool for neurological deficits such as MCI [23].
5. Wisconsin Card Sorting Test (WCST). The participants will be given a deck of 64 cards and will be asked to sort the cards by shape, color, or number. The number of errors made and the number of successful sorted categories (maximum of 6) will be recorded. Participants will receive feedback whether the card sorted is “correct” or “incorrect”; after completing 10 consecutive sorts, the participants will switch to the next sorting category, and this process will be repeated until all the cards in the deck have been sorted. This method is frequently used as a measure of mental switching and response preservation [24].
6. Stroop Test. The participants will be asked to name the color but not to read the word on a card. A color-word score will be given based on the total correct identification of the correct color within 45 s. The Stroop Test is a measure of how the brain process interfering signals. This test measures attention, inhibition control, and response inhibition [24, 25].

### Participant timeline {13}

**Fig. 3:**
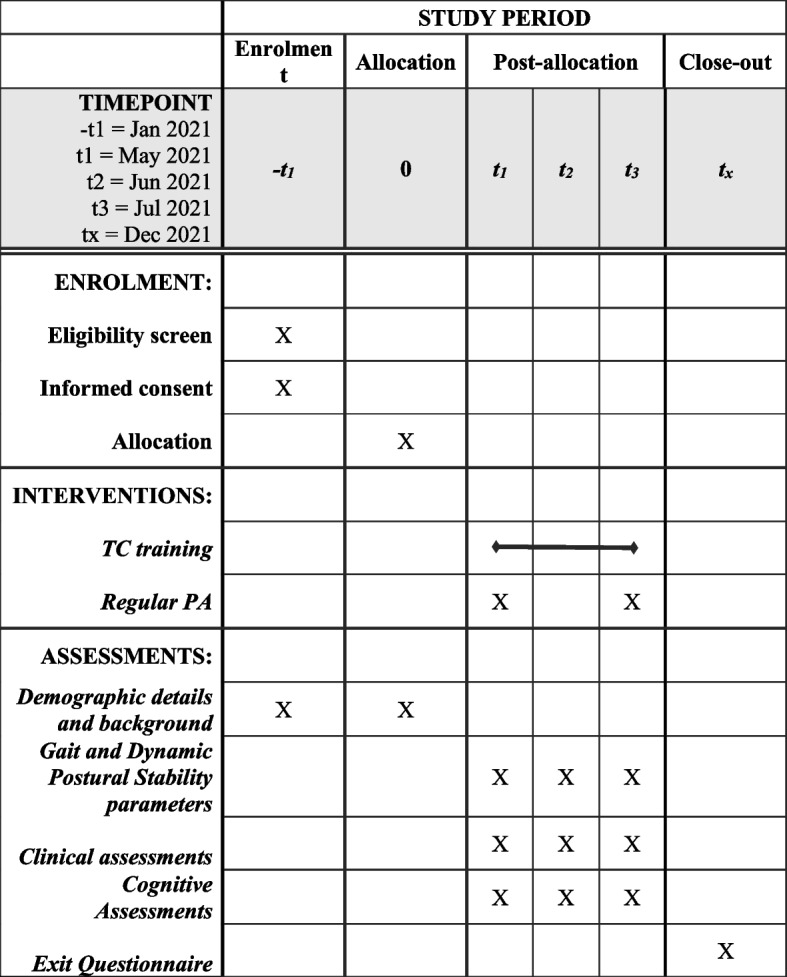
Schedule of enrolment, intervention, and assessment for proposed TC intervention study involving those with Parkinson’s disease

See the participant timeline in Fig. 3.

### Sample size {14}

The estimated number of participants to achieve the study’s objectives is 40, calculated to attain a 93% statistical power in the difference between two means of the independent variables for testing hypotheses with an alpha level of 5% and a minimal clinical important difference (MCID) of 1.13. This estimation is based on the calculation of the minimal clinical important difference that was determined based on the values obtained in past published studies [13, 26–28]. Considering a 10% of dropout rate of the participants recorded from a previous 24-week TC intervention study for those with PD [13], a 5% dropout rate is expected.

### Recruitment {15}

Participants with PD will be recruited in the city of Ottawa and greater Ottawa area, Ontario. All participation in the TC intervention and PA routine will be on the basis that the first randomly allocated 20 participants will be considered for participation in each group. The recruitment of the participants will be obtained through three sources as follows: (1) referrals from local medical clinics (i.e., physician or neurologist offices); (2) local Parkinson’s support groups that are associated with *Parkinson’s Canada*; and (3) bulletin board, newsletters, or postings in the hospital.

## Assignment of interventions: allocation

### Sequence generation {16a}

Potential participants will be screened on the telephone by a research assistant. Participants who are willing and eligible to participate will be randomly assigned in a 1:1 ratio to TC group or PA group by using a concealed block-randomization procedure, stratified by age and sex.

### Concealment mechanism {16b}

The block randomization design will use permuted block sizes of 4 and 6 with the participant assignments placed in opaque, sealed, and serially numbered envelopes and individualized for each participant.

### Implementation {16c}

The randomization schedule will be generated by the data analyst, who will deliver this document in a sealed envelope to the research assistant responsible for assigning the participants to the two groups.

## Assignment of interventions: blinding

### Participants to be blinded {17a}

Changes in the participants’ gait and postural stability and cognitive performance will be evaluated based on their individual baseline scores. This process will ensure that the effects of the two intervention programs are examined instead of the differences in the individual performance or expectations of the participants. The intention and goals of the study will be fully disclosed at the end of the participants’ intervention period.

### Procedure for unblinding if needed {17b}

N/a.

## Data collection and management

### Plans for assessment and collection of outcomes {18a}

A 10-camera Vicon motion analysis system (two Vicon Vantage and eight Vicon Bonita optical cameras, Oxford Metrics, Oxford, UK) with a sampling rate of 200 Hz will be used to capture the participants’ gait and obstacle crossing. Prior to data collection, 45 reflective markers (14 mm diameter) will be placed on the participants’ body landmarks according to a modified Helen Hayes marker set. Ground reaction forces will be collected using four force plates recorded at 1000 Hz (two Kistler models: Kistler Instruments Corp, Winterthur, Switzerland; two Bertec models: Bertec Corporation, Columbus, OH, USA). The force plates will be embedded in the middle of an 8-m-long walkway. The participants from the TC and PA (control) groups will be required to visit the University of Ottawa’s Human Movement Biomechanics Laboratory on three laboratory sessions at baseline and at 6- and 12-week post-intervention. The data collection session will consist of preliminary screening questionnaire, gathering of anthropometric data, 3D motion capture during obstacle crossing and walking, and neuropsychological assessment of executive functioning.

#### 3-D motion capture

1. Gait performance test: Participants will be asked to walk along the 8-m-long walkway at their self-selected comfortable speed during which their movement will be recorded using 3D motion capture for the analysis of their 3D kinematics and kinetics characteristics of the gait. Walking performance will be used as a baseline condition for comparison with obstacle crossing, because it is a simple task to perform and can provide some indications to the participants’ overall health and functional status. Five successful walking trials will be collected.
2. Obstacle crossing test: Participants will perform obstacle crossing in the laboratory for the analysis of their dynamic postural stability and gait performance. An obstacle composed of two upright upholders and a crossing plank with a height of 20 cm will be placed in the middle of the walkway, where the force platforms are embedded. This plank has been made easily collapsible to keep the participants safe to prevent fall. Five successful obstacle-crossing trials will be collected.

#### Neuropsychological assessment

The three following neuropsychological assessment tools were used to examine executive functioning: Trail Making Test Part B (TMT-B), Wisconsin Card Sorting Test (WCST), and Stroop Test. These three neuropsychological assessment tools are commonly used to measure executive functioning in aging research and have been recommended as a popular screening tool for the assessment of mild cognitive impairment [29, 30].

1. TMT-B: participants will be provided with a set of numbers and letters and will be asked to connect a line from a number to a letter in ascending order. The time that it takes the participant to complete this task will be recorded.
2. WCST: participants will be given a deck of 64 cards and will be asked to sort the cards according to shape, color, and number. The number of errors made and the number of successful sorted categories (maximum of 6) will be recorded. Participants will receive feedback about sorting the cards as “correct” or “incorrect”. After completing 10 consecutive sorts, the participants will switch to the next sorting category and this repeated until all the cards in the deck have been sorted.
3. Stroop Test: participants will be asked to name the color but not to read the word on a card. A color-word score will be given based on the total read in 45 s.

For the 3-D motion capture, the data will be collected and analyzed using Vicon’s *Nexus* software (v. 2.8.1) and tabulated in MATLAB (v. 7.1. Release 2006a) to obtain the temporal and spatial parameters. The functional and neuropsychological assessment measures will be gathered and stored both as hard and digital copies for further data interpretation and analysis. Descriptive analysis of the movements will be first conducted. The completion of all trials will be checked, and missing trials will be checked. For the 3D motion capture data of the participants’ movement, the data will be digitized, filtered, and processed. All *Nexus* software calculations will be derived from the Plug-in-gait model (Vicon Oxford Metrics, Oxford, UK). For each trial that will be collected, the data will be normalized to one full gait cycle. One gait cycle is defined as the succession of repeated foot-strike to the next foot-strike of the same foot. The primary outcomes will be normalized by the participants’ body height and weight.

### Plans to promote participant retention and complete follow-up {18b}

Participants in the TC group will be asked to partake in three 1-h TC class per week for 12 weeks. This duration and frequency of practice have previously been used in past TC studies for this population to achieve the desired beneficial effects on postural stability and movement capacity [13, 31]. The selected intensity, duration, and frequency of the training are appropriate to meet the demands of a low-to-moderate level of exercise classification.

To mitigate any feelings of physical discomfort or muscle fatigue, we will instruct the participants to warm up prior to the training and perform each TC form at their preferred comfortable speed. The participants will be allowed to rest or take breaks when they prefer. Based on our previous conducted research in TC biomechanics and intervention, practicing TC is safe to seniors [15, 16, 18, 32]. However, a participant that feels any discomfort may withdraw from the study at any time for any reason.

The safety of the participants in the community center and laboratory settings is a primary consideration. At all times during training session or laboratory testing, both the TC instructor, PI, and/or a research assistant(s) will be present to monitor the performance and abilities of each individual participant. Participants who are deemed to be at risk of falling will be provided with a spotter, who will ensure the safety of the participant. Monitoring by the PI, instructor, and/or research assistant will help to prevent accidental trips and falls. During the TC session, the participants’ progress in the TC program will be monitored. To encourage compliance in the program, the PI will confer with the participants’ daily activity log of their TC practice to see if they are achieving the weekly goals and objectives for the class.

### Data management {19}

The data, which include the completed informed consent form, testing notes, electrical testing data, and printed testing data, will be carefully filed and securely stored in the researcher’s office at the University of Ottawa. The computer in the researcher’s office will also be locked to limit the access. The data will be encrypted and stored in the password-protected and password-encrypted computer of the researcher. The computer will be locked to limit the access. A number code system will be employed during the data collection, analysis, and reporting. In addition, only the researchers can access the number code system in relation to the data obtained from the participants (e.g., electronic data, the master sheet). The master sheet that links the participants’ identification to their number code and other data documents will be kept in a locked filing cabinet of the PI’s office.

### Confidentiality {27}

Information from the study will be held in strict confidentiality and will not be used except for research purpose. The information collected in the study will be coded with anonymity. Any published results will be presented with complete anonymity. Any personal (e.g., address and telephone number) and testing data will be kept in a separate file folder, accessible only by the researchers responsible for the project. The data will be conserved for a period of 5 years after the time of publication. After the time of publication, all the electronic data will be permanently deleted from the computer. The printed data file will be shredded.

### Plans for collection, laboratory evaluation, and storage of biological specimens for genetic or molecular analysis in this trial/future use {33}

N/a.

## Statistical methods

### Statistical methods for primary and secondary outcomes {20a}

Statistical analysis will be performed using SPSS 20.0 and Microsoft Excel 2019 software. Each of the measures will be expressed as mean ± standard deviation. If a measure is not normally distributed, the median and IQR will be calculated to characterize the non-normal distribution. The stratification factors (i.e., age and gender) will be accounted for by an appropriate adjustment technique, such as ANCOVA or multivariable regression analysis. Analysis will be performed to the measures obtained from baseline and at 6 and 12 weeks after TC training. Then, the data between the TC and PA groups will be compared. Two-way analysis of variance (group × time) with repeated measures will be used for examining the effect of TC training on the primary and secondary outcomes. The participants will be analyzed based on their initially allocated groups via randomization, regardless of whether they adhere or if they received the allocated intervention. Statistical significance will be set at *p* less than 0.05.

### Interim analyses {21b}

N/a.

### Methods for additional analyses (e.g., subgroup analyses) {20b}

No planned subgroup analyses were involved in this study. Gender differences are anticipated to be present, but these differences are not one of the primary outcomes of interest or one of the effects of TC training. We do not anticipate any gender effects to remarkably contribute to the effects of TC training. Efforts were made to ensure that only the effects of TC training are examined, by making the intervention groups comparable, setting a strict age range for the participants’ age, and using the individual’s baseline scores for comparisons with their post-training evaluations. Accordingly, we will strictly examine the potential mechanisms and training effects of TC for symptoms related specifically to PD.

### Methods in analysis to handle protocol non-adherence and any statistical methods to handle missing data {20c}

All data will be thoroughly checked for completeness. Should data be missing from any of the gait or obstacle crossing trials, every effort will be made to locate it first before those cases with the missing data will be dealt with using multiple imputation.

### Plans to give access to the full protocol, participant-level data, and statistical code {31c}

Public access will be granted to the full protocol, participant-level dataset, and statistical code upon request.

## Oversight and monitoring

### Composition of the coordinating center and trial steering committee {5d}

The thesis (or steering) committee of the PhD candidate, who will be responsible for conducting and overseeing this study, will guide and provide feedback to develop and refine the details of the study. The thesis committee includes Drs. Denis Prud’homme and Youlin Hong. This committee will ensure that the quality of the candidate’s work meets all academic regulations and requirements for completion of the candidate’s degree. The committee will review the candidate’s work for publications, travel applications, and application funds for conferences and seminars.

### Composition of the data monitoring committee, its role and reporting structure {21a}

A data monitoring committee is not needed, because this role will be fulfilled by the thesis (steering) committee.

### Adverse event reporting and harms {22}

Participants will be allowed to take breaks or rest whenever they prefer. If the participants feel physically exerted, they will be allowed time to rest and water or other forms of rehydration methods would be provided to them. Prior to the performance of the TC routine, the participants will go through a proper warm-up that will involve some basic (light) stretching, breathing, and warm-up movements for the upper and lower extremities. This activity would help mitigate any undesired muscle fatigue, discomfort, or soreness. To reduce the risk of falls or related injuries, the TC instructor, and/or the researchers will be available to monitor the performance of each participant. For participants that are deemed at a higher risk of falling, a spotter will be provided upon either request by participant or if it is evident to the instructor/PI that a spotter is needed. All serious injuries/harm resulting from either the laboratory testing or TC intervention and PA engaged by the participants will be reported to the PI and Research Ethics Board (REB) of the University of Ottawa.

### Frequency and plans for auditing trial conduct {23}

Frequency and plans for auditing trial conduct will not be conducted unless required by potential funding agencies.

### Plans for communicating important protocol amendments to relevant parties (e.g., trial participants, ethical committees) {25}

Considering the recent COVID-19 situation, any important protocol modifications to the investigators, REB, trial participants, trial registries, journals, and steering committees will be communicated accordingly.

## Dissemination plans {31a}

This research protocol could lead to the development of an innovative balance and gait training program that is tailored to individuals with PD. Other training or exercise programs can be modeled from the proposed research protocol in addition to aid, assist, or guide healthcare professionals or therapist in their development or design intervention programs for PD. By examination of the biomechanics of challenging locomotion, obstacle crossing, and TC training impact on gait and posture in the population, it could add understanding to the mechanisms of TC training. This research will help guide the development of more effective exercise programs for health promotion and fall prevention that is not restricted to the target population alone. The work outputs may contribute to multiple publications, conference submissions, and information sessions in support groups for those with PD. This work will satisfy the requirements of a PhD degree in Human Kinetics.

## Discussion

Different types of exercise intervention programs have been developed with limited effectiveness towards treating the movement symptoms related to PD, namely gait disorders and postural instability. Resistance training can help improve strength after a 10- to 12-week period [33, 34], but only in certain groups of muscle, and are ineffective towards training overall postural control and balance. Considering the heterogeneity of the intervention and study outcomes used in these resistance training studies [35], the associated risks of injury, increased level of intensity, and longer duration of resistance training programs required to improve posture and gait are problems for those with PD. Treadmill training for 12 weeks showed an improvement in walking distance [26]. However, the operational costs needed for a body harness or specialized equipment and assistance from a trainer or physical therapist make this form of training not feasible as a long-term option to manage PD. Balance-specific training programs cannot improve all aspects of balance or reduce the risk of falls, mainly because these programs rely on different exercises and activities to retrain balance and posture, with varying levels of effectiveness [36, 37]. Strength, balance, and coordination are some aspects of postural stability that need to be improved, but such programs for PD need to be highly complex and include several multi-modal exercises.

TC, as a “mind–body” exercise, has been applied to manage PD-related symptoms and has increased in popularity because of its rehabilitative potential. TC training shares many features as rhythmic exercises such as tango and ballroom dance that focus on the timing of the participant’s foot placement, whole body coordination, and awareness and attention to their own and partners’ movement [38]. TC is a form of martial art that has been recommended by the *American Geriatric Society* and *British Geriatric Society* for fall prevention in the elderly [39]. However, these activities are only general recommendations that are provided without the proper training protocols and guidelines for those with PD who wish to use TC as a form of therapy or exercise to maintain their physical health and wellbeing. Previous TC programs for PD have demonstrated beneficial effects on posture, gait, and mobility, but limited information is available about the selection basis of TC movement. The mechanisms in the beneficial effects of TC training on gait and postural stability for people with PD are unknown. MRI results have provided support to the beneficial effects of TC practice on brain structure and cognition function [40]. More research on TC intervention on gait, postural stability, and cognition function would improve the understanding of the effects of practicing TC in PD and to access its role and safety as an effective form of exercise to improve quality of the life of this population.

The proposed study protocol is unique and novel, because firstly it is specifically and purposefully designed for PD people for improving their postural stability and gait performance. Furthermore, the biomechanics characteristics of seven TC movements have been understood that forms a foundation for the understanding of the mechanism of TC training. Lastly, with the future development of multimedia materials, tablet, and mobile phone APP’s, the TC intervention program would become more feasible and practical. This protocol could lead to the development of an original and innovative balance and gait training program that could help individuals with PD. The 3D biomechanical analysis of the gait and obstacle crossing in the two groups will enable us to understand the effect of TC training on movement and postural control and how this relates to walking performance and obstacle crossing. This study can serve as a model for healthcare professionals or therapists when designing or developing future intervention programs for PD. The information obtained from the biomechanical analysis of gait and posture in the population would enhance our understanding of the effects of exercise on PD and how to apply exercise in the management of PD.

One possible limitation of the study is that although TC has been gentle on the body, participants with PD may have difficulties following all demonstrations and instructions from the TC instructor. The participants are expected to achieve the necessary level of intensity and duration that would normally be experienced during a light to moderate-intensity exercise. However, their underlying physical and health conditions will need to be carefully monitored by the TC instructor at each TC session. The participants’ progress will be tracked by the researchers, but they will not be challenged beyond their physical and mental limits during practice.

In conclusion, the proposed study will improve the understanding of the effects of TC training among those with PD by using a biomechanical analysis to explain the potential changes in postural stability and gait. The knowledge about the effects of TC training, particularly in gait, postural stability, and cognition, would provide an exercise option for the management of PD and improve the quality of life of this population.

## Trial status

This study is in the early stages of planning as of Aug 2019. Application of the human ethics approval has been submitted and approved as of Oct 2019. Participant recruitment began in Oct 2019. The study will start in May 2021 and will be completed by Dec 2021.

## Supplementary Information

**Additional file 1. **Consent form.

## Data Availability

The PI will have access to the final trial dataset; there are no agreements that may limit such access for the investigators.

## Abbreviations

A-P: Anterior–posterior
COM: Center of mass
COM-COP: Center of mass and center pressure
COP: Center of pressure
H&Y: Hoehn and Yahr
MCI: Mild cognitive impairment
M-L: Medial–lateral
MoCA: Montreal Cognitive Assessment
PA: Physical activity
PD: Parkinson’s disease
TC: Tai Chi
ROM: Range of motion
SLO: Single-leg stance test with eyes open
SLC: Single-leg stance test with eyes open closed
TUG: Timed Up and Go Test
TMT-B: Trail Making Test Part B
UPDRS-III: Unified Parkinson’s Disease Rating Scale- motor examination
WCST: Wisconsin Card Sorting Test
3-D: Three-dimensional
