# Supplementary material for: Effects of a biomechanical-based Tai Chi program on gait and posture in people with Parkinson’s disease: study protocol for a randomized controlled trial
Source: Trials. 2023 Jun 30;24:241. doi: 10.1186/s13063-023-07146-x (PMC10311900; doi:10.1186/s13063-023-07146-x)
Supplement: Supplementary file 1 — Additional file 1. Consent form. [file 13063_2023_7146_MOESM1_ESM.docx]

**Consent Form**

**Title of the study:** The Effects of Tai Chi Intervention on Postural Stability and Gait in People with Parkinson's Disease

**Principal Investigator**: Jing Xian Li ____________________, PhD

Associate Professor

School of Human Kinetics, Faculty of Health Science

University of Ottawa

200 Lees Avenue, Building E (room 020)

(613) 562-5800 ext. 2547

**Co-Investigator:** Nok-Yeung Law ____________________, M.Sc.

PhD Candidate

Human Movement Biomechanics Laboratory

University of Ottawa

200 Lees Avenue, Building E (room 020)

(613) 562-5800 ext. 7358

nlaw098@uottawa.ca

**INVITATION TO PARTICIPATE:**

I am invited to participate in the above-mentioned research testing conducted by Dr. Jing Xian Li of the School of Human Kinetics, Faculty of Health Sciences, University of Ottawa. The research is entitled “The Effects of Tai Chi Intervention on Postural Stability and Gait in People with Parkinson's Disease (PD).” The purpose of the research is to study the effects of tai chi intervention on gait and postural stability in those with PD.

**ELIGIBILITY:**

To be able to participate in this study, I must be between ages of 50 to 75 years. I must be diagnosed with Parkinson’s disease, severity ranging from stage 1 to 3 on the Hoehn & Yahr scale. I must not have any motor fluctuations as measured by the Unified Parkinson’s Disease Scale, motor sub-score (UPDRS-III). I must have stable medication use and be able to stand and walk independently. I must be available to attend or engage in physical activity 3 times/week for a 12-week period. I must not be enrolled in any other behavioral, pharmacological study or instructor led exercise programs during the duration of the study. I must not demonstrate mild cognitive impairment (mini-mental state examination < 24), have poorly cardiovascular disease symptoms during moderate exercise, poorly controlled hypertension, and any debilitating conditions that would prevent myself from engaging in physical activity for at least 60 minutes.

**PROTOCOL:**

I will randomly be assigned to either groups: tai chi or physical activity.

If I am enrolled in the tai chi group, I will be taught tai chi in a class size of 5 to 12 students offered in the laboratory or community center, by a certified tai chi instructor. Each tai chi class will be 60 minutes, offered 3 times per week. I am encouraged to attend at least two sessions per week for up to 12-weeks. To facilitate learning of the movements, I will be given a hard copy of the illustrated 6-form tai chi program or a 5 to 10-minute demonstration video with the instructor.

If I am enrolled in the physical activity group, I will be required to engage in at least 60-minutes (3 times per week for 12 weeks) of regular physical activity on their own that will be documented in an activity log or journal. Some types of activities I will be allowed to engage in will include walking, jogging, stair climbing, or light stretching.

**PARTICIPATION:**

I will attend three sessions of test at the Human Movement Biomechanics laboratory, 200 Lees Avenue, University of Ottawa. The test will be at the beginning, 6, and 12 weeks after commencing either exercise programs, such as tai chi or regular physical activity. I will have to complete and return a form of my exercise or physical activity practicing activity every month for monitoring purposes. For my neuropsychological assessment, I will allow the researcher to assess my cognitive functioning using three commonly used neuropsychological assessment tests. During my movement assessment, I will wear shorts and a shirt that will be provided for motion capture and have small reflective markers placed on chosen anatomical landmarks on my body. I will have pairs of electrodes attached on my limbs for electromyography analysis. My body weight and height will be measured. My movement while walking and during obstacle crossing will be recorded using a 3-dimensional video recording system. I will perform 5 trials for each of type of movements, respectively. The data collection session will last about 2 hours.

**RISKS:**

There are no risks involved. The researcher has assured me that I will have enough time to practice crossing over the obstacle and warmup before formal data collection and I may request to stop at any time if I need to rest. In any case I am not able to keep my balance under these conditions I can withdraw from the study. The researcher has assured me that if there is any lingering discomfort, I can withdraw from the study at any time.

**DISADVANTAGES OF PARTICIPATING:**

I understand the primary disadvantage of participation will be the time required to complete and to perform data collection.

**BENEFITS:**

I will not benefit directly from my participation. However, I will gain a better understanding on my exercise engagement and physical activity performance. I will be able to learn how my participation in an exercise program and/or regular physical activity affect my physical capacity and wellbeing. My contribution in this research will be beneficial for the understanding of the effects of exercise in those with Parkinson’s disease.

**CONFIDENTIALITY AND CONSERVATION OF THE DATA:**

I understand that the data collected from me will be used only to serve the research’s purpose and data might be used in student theses and my confidentiality will be ensured. I will be assigned an anonymous identification code that will be used throughout the research. The data (completed consent forms, testing notes, and measurement recordings in paper form) will be stored in a locked filing cabinet in the office of the researcher at the University of Ottawa. The electronic data will be kept in the computer of the researcher and will be password protected. Only the researchers will have access to the data. I understand that any published results will be presented with complete anonymity.

The data will be conserved for a period of 5 years after the time of publishing the research results, at which time the electronic data will be deleted, and the printed data will be shredded.

**ANONYMITY:**

Anonymity will be assured in the following manner: a code number system will be employed during the data collection, analysis, and reporting. I will not be identified in any reports or publications.

**COMPENSATION:**

I will receive $15.00 at the beginning of the session of evaluation of my postural stability and movement capacity to help cover my travel and parking costs. After the study has begun if I choose to withdraw, I can still receive the compensation. A simple meal will also be provided with to a maximum of $15 if the biomechanics assessment goes over regular mealtime (lunch or dinner).

**VOLUNTARY PARTICIPATION:**

Participants who choose to withdrawal from the study will also be given the opportunity to withdraw their data.

I understand that I may ask questions regarding this study at any time and they will be answered. These questions may be addressed to Dr. Jing Xian Li (613.562.5800 ext. 2547)

**MORE INFORMATION ABOUT THIS STUDY:**

If I have any questions with regards to the ethical conduct of this study, I may contact the Protocol Officer for Ethics in Research, University of Ottawa at Room 154, Tabaret Hall 550 Cumberland Street, Ottawa, ON, K1N6N5, or (613) 562-5387 or [ethics@uottawa.ca](mailto:ethics@uottawa.ca).

**CONSENT:**

I declare that I understand this project, the nature and degree of my participation and possible disadvantages and risks listed in this consent form. I have had the opportunity to ask all my questions concerning the different aspects of the study and have received responses to my satisfaction.

**I voluntarily agree to participate in this study.**

**I agree that Dr. Jing Xian Li can keep my name and telephone number for contact me for any future study that she may be carrying out.**

**Yes _________ No_________**

There are two copies of this consent from one of which is for me to keep.

__________________________

Name of Participant

__________________________ __________________________

Signature of Participant Date

__________________________ __________________________

Signature of Researcher Date
